# American Medical Association

**Published:** 1877-06

**Authors:** 


American Medical Association.—The twenty-eighth annual meeting of this body was held, as we learn from our exchanges, in Chicago on the 5th instant, and was opened by an address from Dr. J. Marion Sims, the retiring president. Dr. Bowditch, of Boston, the President elected at last meeting, was then introduced. Dr. N. S. Davis delivered the address of welcome, after which discussions were had on the following papers from Sections:

Administration of Quinine in Pneumonia, by N. S. Davis; Extirpation of the Uterus, by Dr. Kimball; Treatment of some Diseases of the Female Urethra, by Dr. W. H. Byrd; Certain Operations for Vesico-Vaginal Fistula, by Dr. Bozeman; Value of Extension in the Treatment of Fractures of the Femur, by Dr. Hodgen; Relation of Spiritualism to Medical Jurisprudence, by Dr. John P. Gray.

*Second Day's Proceedings.*—In Dr. Squibb's address on Revision of the Pharmacopœia, reference was had to the pamphlet of Dr. Wood opposing the revision by the American Medical Association. Dr. Squibbs urges it, notwithstanding the objections made by Dr. Wood.

The following papers from sections were read and discussed: Transmission of Enteric Fever by Dairy Milk, by P. G. Robinson; Neglect of Obstetrical Forceps, by Dr. J. P. White; Clinical and Meteorological Records, by Dr. N. S. Davis; Congenital absence and imperfect development of the Uterus, by Dr. Marcey; Strictures of the Urethra from Masturbation and its Pathological Significance, by Dr. S. W. Gross; Medio-Bilateral Lithotomy, by Dr. W. T. Briggs; Treatment of Fractured Ribs by Extension and Expansion of the Thorax and Retention by Plaster of Paris Bandage, by Dr. Lewis A. Sayre; Do Facts Justify the Recognition of Moral Insanity as a Distinct Form of Mental Disease? by Dr. R. J. Patterson.

*Third Day's Proceedings.*—The Treasurer reported a small surplus, but unusually heavy expenses during the past year.

The Committee on Publication urged the employment of a medically educated stenographer.

The Committee on Prize Essays reported the reception of only two, neither of which was worthy of a prize.

The question of Revision of the Pharmacopœia was discussed again, but finally the whole subject was tabled.

The following officers were elected: President, T. Y. Richardson of Louisiana; Vice-Presidents, Drs. White of New York, Green of Illinois, Russell of Connecticut, and Dunlap of Ohio.

The report of Drs. J. J. Woodward, U. S. A., and Edward Seguin, on the progress of uniformity in the means of observation and record, was submitted.

A resolution was adopted favoring the passage of the "Morrison Bill," now before Congress, repealing the tariff on quinine.

Buffalo was selected as the next place of meeting, on first Tuesday in June, 1878.

